# Room-Temperature Fiber-Coupled Single-Photon Sources based on Colloidal Quantum Dots and SiV Centers in Back-Excited Nanoantennas

**DOI:** 10.1021/acs.nanolett.3c03672

**Published:** 2024-01-02

**Authors:** Boaz Lubotzky, Alexander Nazarov, Hamza Abudayyeh, Lukas Antoniuk, Niklas Lettner, Viatcheslav Agafonov, Anastasia V. Bennett, Somak Majumder, Vigneshwaran Chandrasekaran, Eric G. Bowes, Han Htoon, Jennifer A. Hollingsworth, Alexander Kubanek, Ronen Rapaport

**Affiliations:** † Racah Institute of Physics, The Hebrew University of Jerusalem, Jerusalem 9190401, Israel; ‡ The Center for Nanoscience and Nanotechnology, The Hebrew University of Jerusalem, Jerusalem 9190401, Israel; § Institute for Quantum Optics, University of Ulm, Albert-Einstein-Allee 11, 89081 Ulm, Germany; ∥ Center for Integrated Quantum Science and Technology (IQst), Ulm University, Albert-Einstein-Allee 11, D-89081 Ulm, Germany; ⊥ GREMAN, UMR 7347 CNRS, INSA-CVL, Tours University, 37200 Tours, France; # Materials Physics & Applications Division: Center for Integrated Nanotechnologies, Los Alamos National Laboratory, Los Alamos New Mexico 87545, United States

**Keywords:** integrated single-photon source, hybrid metal−dielectric bullseye antenna, colloidal quantum dot, SiV-centers, fiber-coupled single photons, quantum key distribution, quantum cryptography

## Abstract

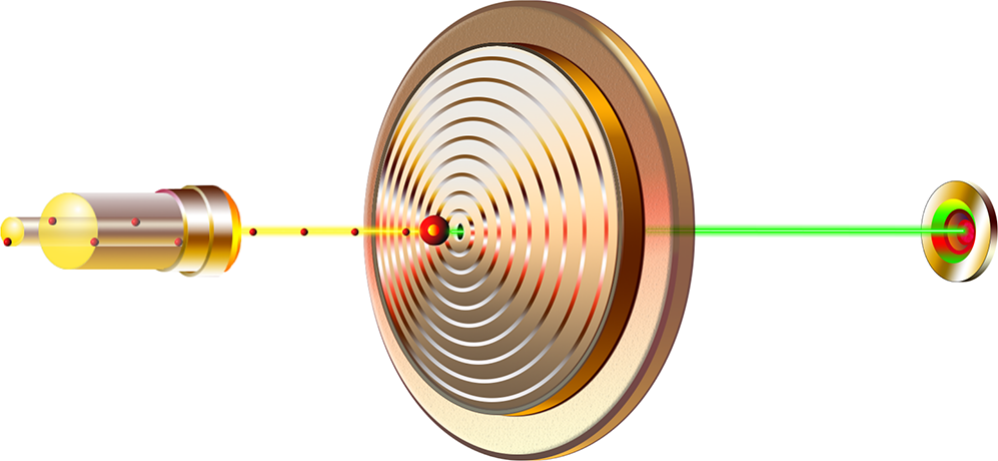

We demonstrate an important step toward on-chip integration of single-photon sources at room temperature. Excellent photon directionality is achieved with a hybrid metal–dielectric bullseye antenna, while back-excitation is permitted by placement of the emitter in a subwavelength hole positioned at its center. The unique design enables a direct back-excitation and very efficient front coupling of emission either to a low numerical aperture (NA) optics or directly to an optical fiber. To show the versatility of the concept, we fabricate devices containing either a colloidal quantum dot or a nanodiamond containing silicon-vacancy centers, which are accurately positioned using two different nanopositioning methods. Both of these back-excited devices display front collection efficiencies of ∼70% at NAs as low as 0.5. The combination of back-excitation with forward directionality enables direct coupling of the emitted photons into a proximal optical fiber without any coupling optics, thereby facilitating and simplifying future integration.

Solid-state-based single-photon sources (SPS) are the key for a host of quantum technologies. They serve as essential building blocks for quantum metrology,^1−3^ quantum networks,^4−7^ and quantum information processing.^8−10^ For the room-temperature (RT) operation of SPS, colloidal quantum dots (CQDs) are now capable of single-photon emission on demand with high brightness, good photon purity, and a tunable spectrum. Color centers in diamonds display a very stable single-photon emission.^4,11,12^ Specifically the negatively charged silicon vacancy center in diamond (SiV) combines a short lifetime and a narrow-band, linearly polarized emission.^13^ However, a major problem of these sources is their isotropic emission, which results in low collection efficiency (CE), severely limiting usefulness. Modifying the photonic environment near the emitter using dielectric or metallic antenna structures can change the angular emission pattern^14,15^ and lead to higher collection efficiencies. Coupling the emitter to high-quality-factor dielectric antennas enables low loss and high CE but is suitable only for narrow frequency bandwidth, which, typically, does not cover the bandwidth of RT emitters and requires complex fabrication. Another possibility is to couple to metallic antenna structures. The combination of low quality factors with low mode volumes enables highly directional emission over a broad spectral range. However, efficient coupling requires a short distance between the emitter and the metal, which produces high nonradiative recombination rates.^15,16^ A possible solution that combines the advantages of dielectric and metallic antennas, such as high directivity over a broad frequency bandwidth with low loss is the use of a hybrid metal–dielectric circular Bragg (bullseye) antenna.^17^ This concept has been utilized in recent years to demonstrate highly directional RT single-photon emission from a single CQD^18^ and from a single NV center in a nanodiamond,^19^ with record-high, near-unity collection efficiencies^20^ combined with a large optical Purcell factor to yield both a high brightness and excellent directionality of single photons.^21^

However, two major challenges remain for the on-chip integration of RT SPS devices: the need for direct coupling of emission into an optical fiber and a strategy for integrating a laser excitation source compactly into the same SPS device. Recently, several works presented a fiber-coupled SPS.^22−28^ These systems remain limited to operation in a cryogenic environment. Furthermore, for network applications, it is desirable to have a monolithic device that contains multiple RT SPSs on the same chip. Such a device should be designed to allow either independently or synchronously addressing and exciting these SPSs and couple their emission to several optical fibers or a single one. This configuration, especially when integrating multiple SPSs into a single fiber, becomes pivotal for quantum key distribution (QKD) networks employing the wavelength division multiplexing method. Relying solely on optical pumping through the collection optical fiber cannot achieve these sophisticated functionalities. Here, we address these challenges by avoiding the need for the single photons and the excitation laser to share the same optical path or the same fiber, paving the way for quantum key distribution network applications.

Our platform consists of an emitter coupled to a hybrid metal–dielectric bullseye nanoantenna having a subwavelength hole at its center. This geometry enables direct laser excitation from the back and facile coupling of emitted photons to either free-space optics or an optical fiber from the front. The approach is versatile, as we demonstrate nanoantenna-SPS devices using two different photon sources: CQD and SiV in nanodiamonds. These are accurately coupled to the nanoantennas using two different nanopositioning methods, dip-pen nanolithography (DPN) and pick-and-place techniques, respectively. Significantly, both devices produce a highly directional forward photon emission with negligible back-leak that results in a CE of ∼70% at numerical apertures (NA) as low as 0.5. Moreover, the combination of back-excitation with forward low NA directionality enables a coupling of the emitted photons directly into a proximal optical fiber without the need for further coupling optics, facilitating and greatly simplifying future integration into envisioned photonic quantum networks.

The design concept^17,18,20^ and fabrication method resemble those of previous hybrid metal–dielectric nanoantenna devices (Figure 1).^20^ The nanoantenna consists of a gold substrate (with a thickness of *H*) with a gold circular Bragg grating (height *l* and period Λ) surrounding a central circle (diameter *D*). The metal component is covered by a transparent dielectric layer of aluminum oxide (height *h*). The geometrical parameters of the antenna were numerically optimized^17,20^ (using 3D FDTD Lumerical simulations) for best directionality of the emission from a dipole emitter placed inside the dielectric layer at the center of the antenna. A different set of parameters is found for different emitter central wavelengths, corresponding to the two different emitters used in this work, namely, CQDs (650 nm) and SiV (737 nm). See Appendix A in the Supporting Information for optimized values. The template stripping method used to fabricate the metallic structure ensures high-quality bullseye antennas.^20,21^

**Figure 1:**
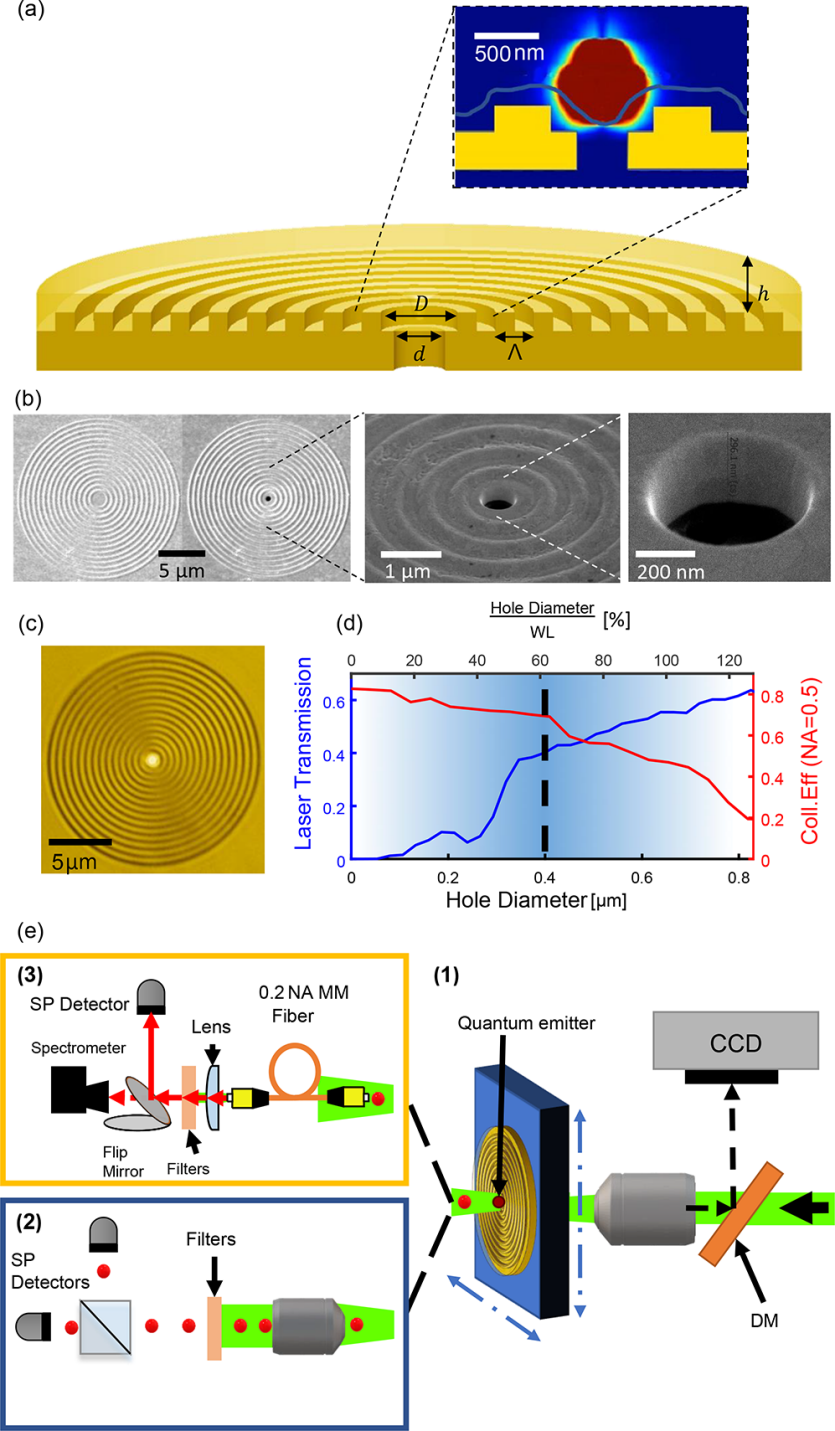
Device schematics and experimental setups. (a) Schematic cross section of hybrid metal–dielectric bullseye structure with a hole at the center with the key geometric parameters: the central cavity diameter (*D*), hole diameter (*d*), groove period (Λ), polymer thickness (*h*), and total metal thickness (*l*). The inset illustrates a close-up of the electric field distribution 3D Lumerical simulation of a dipole source located in the center of the antenna, above the hole. The blue line marks the boundary of the layer of aluminum oxide as measured on a sample using AFM. It can be seen that a hole with a diameter of 400 nm does not allow the transmission of the light emitted from the dipole source. (b) SEM images of the bullseye antenna before and after digging the hole. (c) Microscope image of the bullseye antenna showing light coming from the hole at the center. (d) Laser transmission through the hole and collection efficiency at different hole diameters. The top axis displays the diameter of the hole divided by the wavelength of the emission. The dotted black line marks the actual diameter we used for this study. (e) Illustration of the experimental setup: (1) the back-excitation system with a dichroic mirror (DM) on the laser path for back imaging, (2) the front imaging system, and (3) the fiber coupling system.

Unlike previous designs, before covering the metal with a dielectric layer, a hole with a diameter *d* = 400 nm is drilled at the center using a focused ion beam (FIB), penetrating all the way to the transparent substrate. See Appendix A for details. The antenna is imaged before and after hole drilling (Figure 1b), while white light can be easily detected passing through the hole (Figure 1c). The hole size must be large enough to allow transmission of laser light from the back of the device through the hole,^29−31^ yet small enough to maintain the CE by reducing the transmission of the light emitted from the dipole emitter (Figure 1a). This allows coupling to the forward propagating modes of the antenna. To determine the optimal hole size for the device, we performed calculations based on FDTD simulations. Figure 1d shows the calculated dependence of the laser transmission through the hole and the far-field CE of the dipole emitter (for NA = 0.5) on the diameter of the hole for 532 nm light, which is the longest wavelength we used for excitation. For a shorter wavelength, even more laser excitation power can pass through the hole. As for the CE, we show it for an emitter at 650 nm, which is the shorter of the two central wavelengths corresponding to the two nanoemitter types measured in the experiment. Here again, for a longer wavelength, even less emission is expected to leak back through the hole.^29−31^ The dotted black line marks the size of the hole (*d* = 400 nm) that was chosen, for which more than 40% of the back-excitation is transmitted and the CE for NA = 0.5 is larger than 70%. Before the emitter was placed on the antenna, the sample was covered with a dielectric layer of Al_2_O_3_ using ALD deposition (blue line in the inset of Figure 1a indicates the boundary of the layer of aluminum oxide as measured on a sample using AFM). The thickness of the oxide layer was chosen to optimize collection efficiency for each emitter type. In this way, the device is easily tailored to the particular single-photon source.

Typically,^18,20,21,32−34^ measurements are done in a front excitation configuration, where the optical path of the laser excitation coincides with that of the emission collection. In that case, the same objective is used for both excitation and collection of photons from the quantum emitter. In contrast, in this work, a back-excitation/front-collection scheme is enabled by the unique antenna design. As presented schematically in Figure 1e the laser light is focused onto the sample using a back objective (1) to excite the quantum emitter through the hole in the nanoantenna. A CCD camera imaged the back side of the antenna to align the excitation path. From the front side of the sample, a collection objective (2) or a fiber (3) is used to collect the emission of the quantum emitter. After light passes through the objective or the fiber, dichroic filters are used to filter out the excitation light with the emitter photons reaching the detection arms, consisting of either a spectrometer with a CCD camera or a single-photon detector setup capable of measuring emission lifetime traces or second-order photon correlations. Importantly, the two distinct optical paths allow the excitation conditions (e.g., position, intensity, incoming polarization, and spot size) to be varied separately without changing the collection path. For example, an objective with low NA can be used to enlarge the excitation spot size on the back of the sample, relaxing exact positioning of the excitation spot and allowing several nanoantennas to be excited at the same time without sacrificing the NA of the collection objective. In addition, the optical components can be optimized for the specific wavelength and polarization of the excitation laser without restrictions imposed by optimization of the emission collection. The dual-optical system is thus flexible and suitable for a greater variety of emitters and conditions, as shown below. Beyond these advantages, in this geometry an optical fiber can be used to directly collect the front emission without the requirement that the same fiber also be used to excite the emitter. In contrast, excitation through the fiber necessitates use of very high intensities (due to its low NA compared to high-NA objectives), which adds unwanted noise in the collection of the emitted photons due to strong broad-band autofluorescence of the fiber.

Figure 2 displays the results for a single CQD placed at the center of the nanoantenna. The employed CQDs are CdSe/CdS core/thick-shell nanocrystals, also known as giant CQDs (gQDs). gQDs are characterized by essentially nonblinking and nonphotobleaching behavior at room temperature^35−37^ and in this case were synthesized by a modified continuous-injection procedure,^37,38^ providing a near-unity quantum yield of 80% and monoexponential photoluminescence decay in solution. The high level of stability allows gQDs to provide photons without interruption by fluorescence intermittency or catastrophic failure. A direct-write nanolithography method known as dip-pen nanolithography (DPN) was used to place individual gQDs into the holes of the bullseye structures in Figure 2a. DPN is amenable to scaling^20^ and in previous work demonstrated a 25% success rate for depositing either a single gQD or a small cluster in standard bullseye antennas.^20^ In this work, all depositions resulted in placement of either a single gQD or a cluster, as revealed by preliminary second-order correlation (*g*^(2)^(τ)) experiments applied using a time-gated filtering technique.^20,39^ See Appendix B for details.

**Figure 2:**
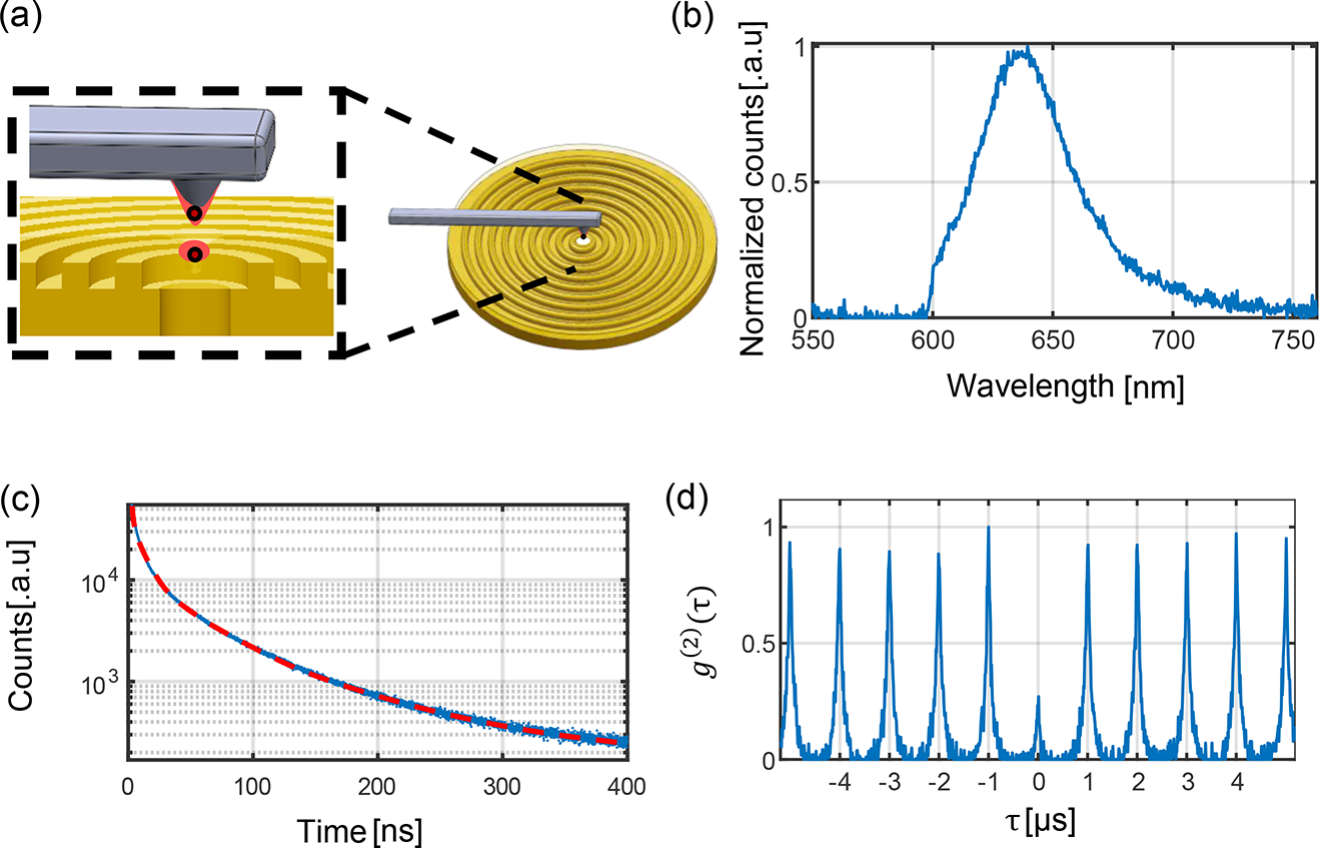
Results from a single QD coupled to a bullseye nanoantenna with a hole at the center. (a) Schematic representation of the placement method used for placing the QD on the hole at the center of the bullseye antenna. (b) QD emission spectrum measurement, performed by back-excitation spectrally filtered in the spectral range between 600 and 750 nm. (c) QD fluorescence lifetime measurement on a semilogarithmic scale, performed by back-excitation. The data exhibit a biexponential decay with components of 89 ns (26%) and 22 ns (74%) identified as the single exciton and trion emission, respectively. (d) Second-order correlation measurement using a Hanbury–Brown–Twiss experiment with time-gated filtering technique, displaying an antibunching of less than 0.5.

Successful through-hole excitation of a gQD positioned in the front side of the nanoantenna by a laser positioned on the backside was confirmed. A representative fluorescence spectrum is shown in Figure 2b. The photoluminescence decay trace is shown for the same gQD in Figure 2c, revealing a biexponential decay. The QDs used in this work exhibit high QY, symmetric emission, and monoexponential PL decay, as we show in Figure S2b, Appendix B in the Supporting Information. As previously reported,^40^ these thick-shell QDs are capable of efficient trion, biexciton, and multiexciton emission, evident under higher pump intensities. A biexponential decay is observed for the single QD, as shown in Figure 2c. This decay retains the slow ∼89 ns exciton component (26%) from the ensemble solution measurement but introduces a new fast component of ∼22 ns (74%), which is consistent with trion emission.

In order to confirm that the emission is from a single gQD, the results of a time-gated *g*^(2)^(τ) experiment^20,39^ was performed (Figure 2d) using nonresonant (λ = 405 nm) pulsed-laser back optical excitation at a repetition rate of 1 MHz. With time filtering of τ_f_ = 60 ns a clear antibunching is observed.

Symmetry-protected, negatively charged silicon-vacancy centers (SiV) stand out from the family of color centers in diamond for their dominant photon emission into the zero-phonon line (ZPL) with a Debye–Waller factor of about 70% as well as spectrally stable and intrinsically indistinguishable optical transitions.^41,42^ The properties persist even when the SiV center is hosted in crystals at the size of visible wavelengths.^43^ Such nanodiamonds (NDs) offer the possibility of increasing the spin coherence times at cryogenic temperatures by modifying the electron–phonon coupling, thereby suppressing orbital relaxation.^44^ Furthermore, the small size enables precise atomic force microscope (AFM) based nanomanipulation for fabrication of hybrid quantum-photonic devices.^45,46^ To place the NDs we use the pick-and-place method, which benefits from a high accuracy of ∼10 nm. The central hole in the bullseye structure eases the placement of the ND due to its topology (Appendix A). An AFM scan and confocal imaging verify ND positioning. Figure 3b shows an AFM scan of the ND placed on the hole and a photoluminescence spectrum for a single antenna-coupled ND was obtained by back-excitation with 532 nm CW laser through the hole, with emission collected from the front (Figure 3d). A short-pass filter (750 nm) was used and is responsible for the decrease to zero of the intensity at wavelengths above 750 nm.

**Figure 3:**
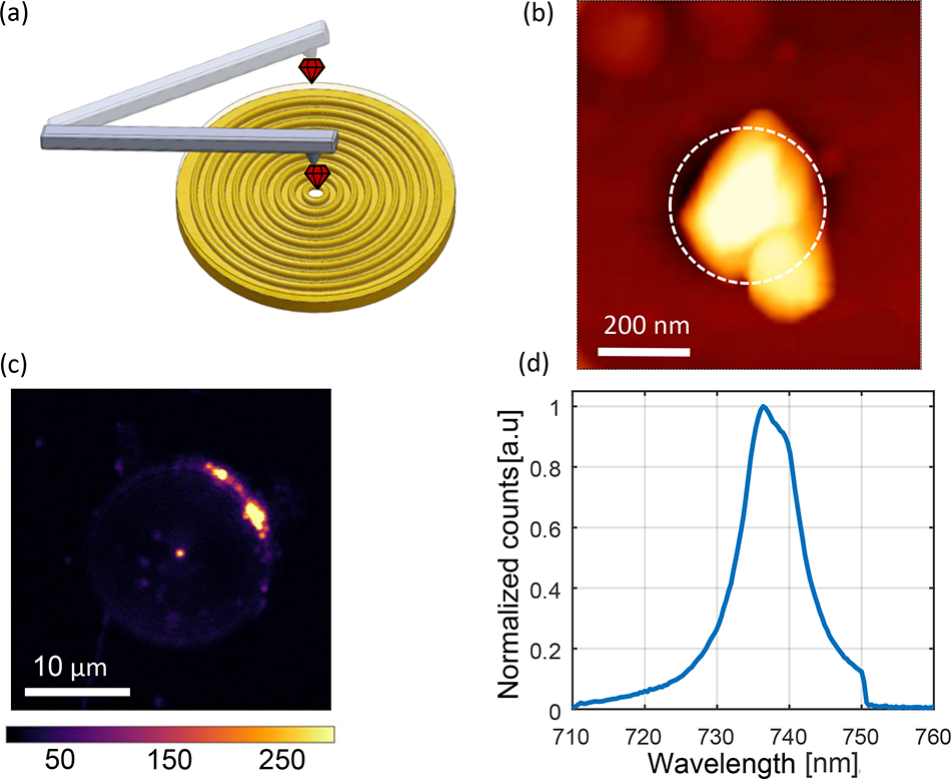
Results from a single ND containing SiV centers coupled to a bullseye nanoantenna with a hole at the center. (a) Schematic representation of the placement method used for placing the ND on the hole at the center of the bullseye antenna. (b) AFM scan showing the ND placed on the hole. The white dashed line indicates the hole. (c) Confocal scan using 532 nm as excitation wavelength of a bullseye antenna showing the emission from the ND located on the hole in the center of the antenna. (d) SiV center emission spectrum measurement, performed by back-excitation. A short-pass filter was placed at 750 nm.

Emission directionality was measured using Fourier-plane PL imaging (Figure 4a; θ and ϕ are the polar and azimuthal angles, respectively) to evaluate the performance of both back-excited devices. The resulting back-focal-plane images (Figure 4b,e) and extracted azimuthally integrated angular intensity distributions (Figure 4c,f) reveal the highly directional emission obtained for each emitter type. Collection efficiency (CE, η) as a function of NA (Figure 4d,g) was obtained by integrating the signal over all azimuthal angles (ϕ) within a collection cone of a given NA relative to the calculated signal for NA = 1, according to
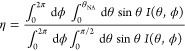
1For each emitter, the experimental CE is compared with an FDTD simulation that incorporates an incoherent summation of single linear dipoles positioned at the center of the device. This approach accounts for both unpolarized CQD emission and the complex emission properties of SiV centers, consistent with the findings in ref (47). For low NAs, the SiV-based device yielded lower directivity compared to both the simulation and the CQD device. We attribute this drop in directivity to the extended spatial distribution of the SiV centers within the nanodiamond, which is large compared to that of the nanodiamond employed in our previous work.^34^ It can be noted that there are small differences in the collection efficiency between the simulation and the experiment. We speculate that the source of these differences is related to the accuracy of the position of the emitter. The hole enhances accuracy in the *XY* plane, but it may also influence the *Z*-axis precision due to its effect on the surrounding polymer layer’s thickness and the exact height of the emitter.

**Figure 4:**
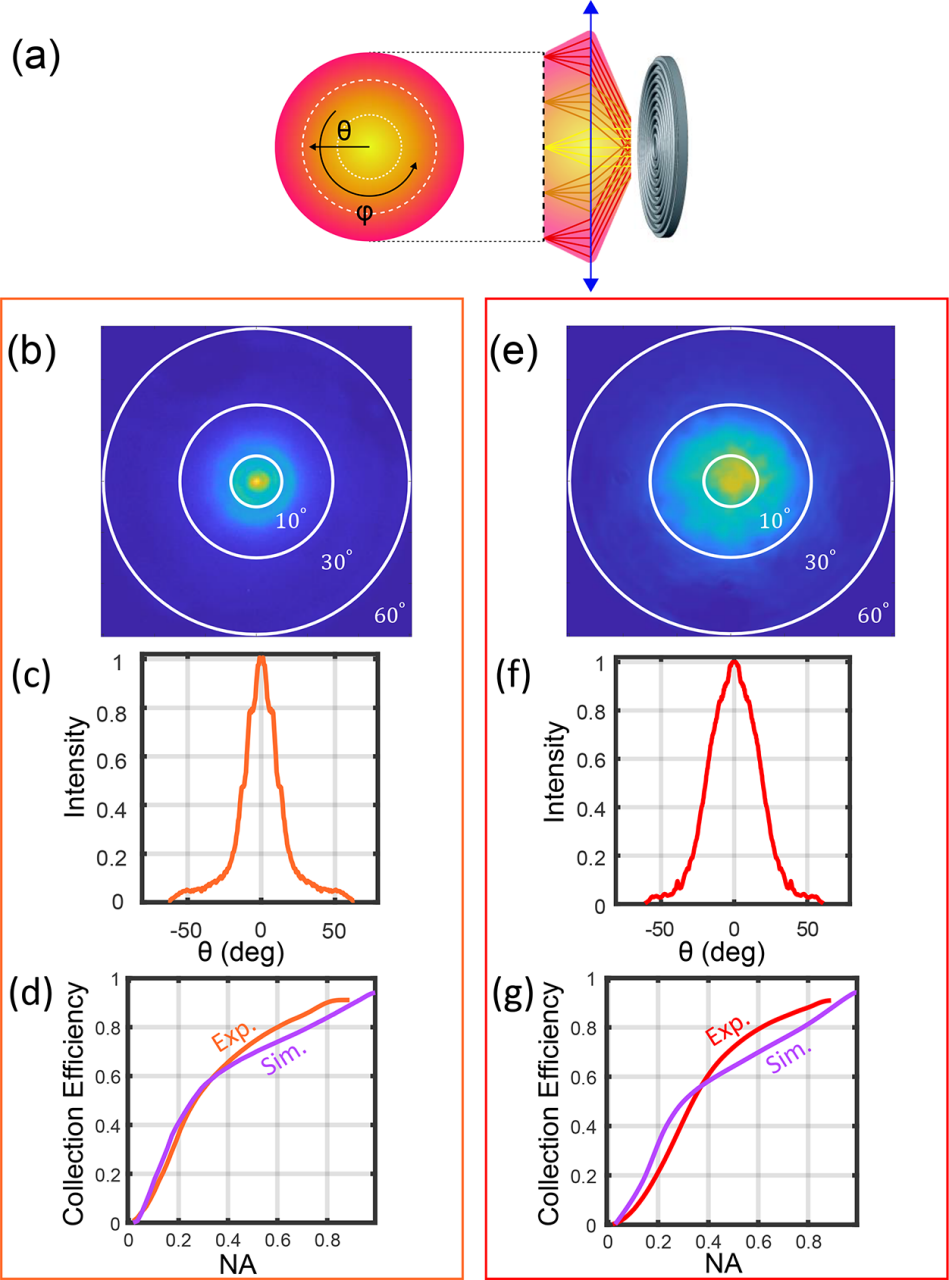
Back focal plane and collection efficiency measurements and calculations of the device. (a) Schematic representation of the back focal plane imaging technique used to measure directionality in this study. The orange frame (b–d) shows results of the device with a single QD, and the red frame (e–g) shows results of the device with a single ND containing SiVs. Back-focal plane image (b, e), azimuthally integrated angular intensity distribution (c, f) and simulated (purple) and measured (red) collection efficiencies for emitter antenna devices.

Toward an integrated photonic device, we demonstrate direct coupling into a proximal optical fiber without the need for complex, bulky optical elements in between. And we do so at room temperature in contrast with prior studies.^22−28^ Here, we employ a gQD-based device and for ease of detection one containing multiple gQDs (Figure S1 in the Supporting Information). The experimental setup, for which the same back-excitation objective is used as in the free-space measurements, is shown (Figure 5a). To optimize device–fiber coupling, a piezoelectric stage was used to align the fiber position with respect to the nanoantenna (Figure 5a). PL decay traces and PL spectra are compared for different excitation and collection configurations (Figure 5b,c). PL lifetimes and the characteristic biexponential decay are unaffected by the mode of collection (Figure 5b)—0.9 NA objective (orange) or 0.22 NA MM fiber (blue). Figure 5c shows a comparison of three intensity-normalized PL spectra obtained via back-excitation/front-collection using a 0.9 NA objective, front-excitation/front-collection with a 0.9 NA objective, and back-excitation/direct 0.22 NA fiber collection. All three measurements display a similar spectrum typical to the gQD (central peak ∼645 nm), proving successful direct-fiber coupling. The PL angular intensity map of the device under back-excitation shows an angular spread approximately corresponding to the NA of the MM fiber. This match in scale likely accounts for successful direct-fiber coupling. To estimate the coupling efficiency into free space and fiber, we conducted a photon-count comparison taking into account different losses of the optical setup (see Appendix D). The 0.22 NA fiber-coupling efficiency was observed to be 9.88 ± 0.48%, approximately 3 times lower than that of free-space 0.22 NA CE (Figure 5e). We attribute this difference to imperfect alignment, mode mismatch between the free space and the MM fiber, and dielectric reflection from the uncoated fiber facet. The apparent spectral broadening in Figure 5c is visible due to plasmon radiation caused by high-intensity excitation that was used for back-excitations (Appendix D) and also partially attributable to MM fiber spectral broadening.^48^ The plasmon emission can be filtered using temporal filtering with pulse excitation as evidenced in ref (17). This result opens a path toward on-chip single-photon devices with direct-fiber coupling integrated with a back-side excitation source.

**Figure 5:**
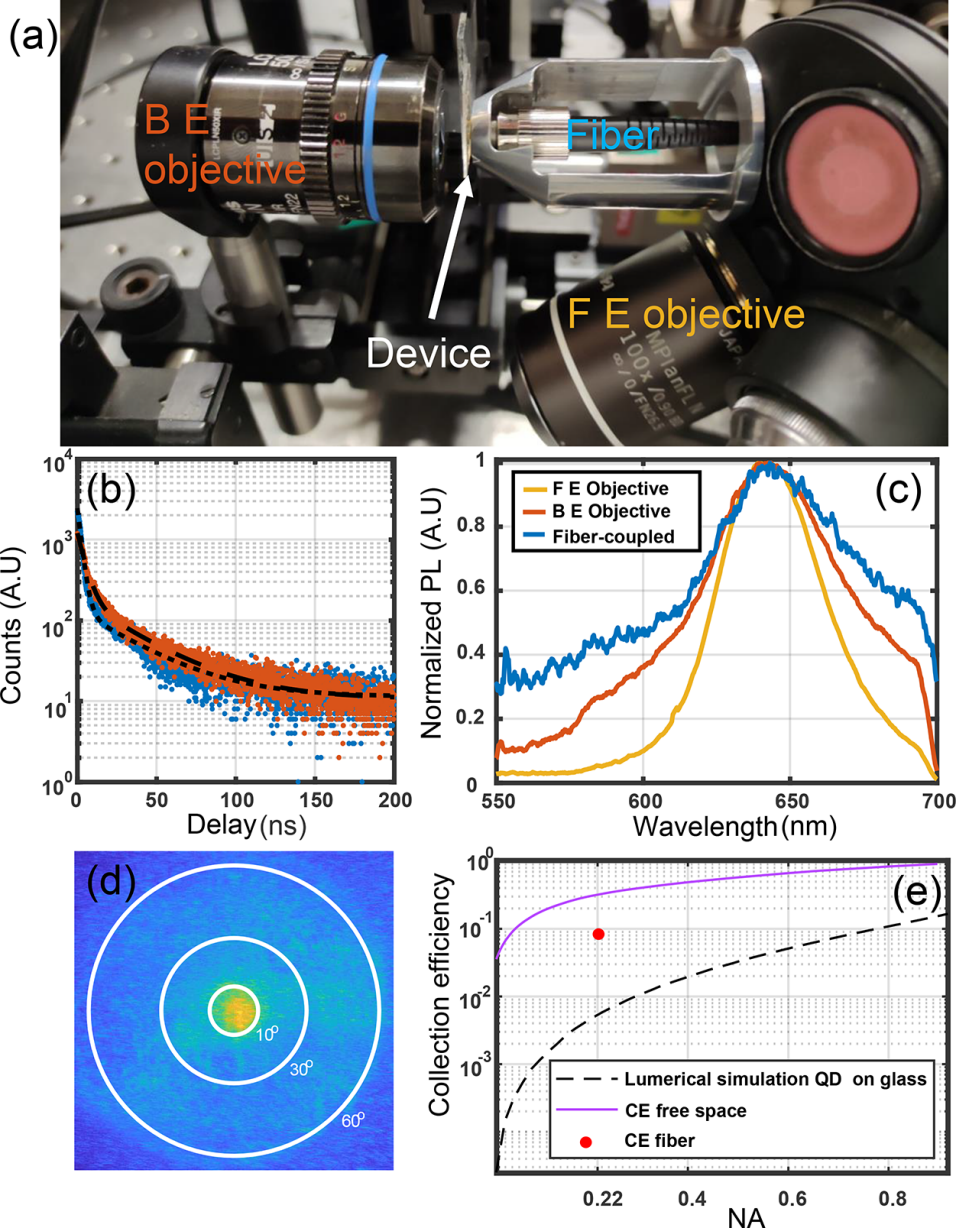
(a) Setup image. (b) Lifetime comparison between an objective collection (orange) and a fiber-coupled (blue) emission. (c) Spectral measurements using three different configurations: front-excitation (F E) in free space (yellow), back-excitation (B E) in free space (orange), and back-excitation with fiber collection (blue). To isolate specific spectral regions, spectral filters were placed at 550 and 700 nm. (d) Back focal plane image of the same device. (e) Comparison of back-excitation collection efficiency (magenta) and QD on glass collection efficiency (black).

We demonstrated the emission and collection of single-photons from a back-excited emitter/nanoantenna device. The efficient back-excitation was coupled with high forward directivity and high collection efficiency in the free-space and coupled fiber. Devices comprising either gQD or SiV-center emitters in the metal–dielectric bullseye antennas with a subwavelength hole at the center were easily accessible using two distinct nanopositioning strategies. GQDs were placed using DPN, which is amenable to scaling and is highly accurate,^20^ while NDs containing SiV-centers were placed using the pick-and-place method, which is also highly accurate and enables multistage position optimization and dipole rotation optimization.^49^ Remarkably, the drop in CE as a result of adding the hole compared to similar devices without a hole and with front-excitation^20,50^ amounts to less than 10% for an NA of 0.5. We attribute this minor drop in CE to emission losses into the hole, as well as less-effective emission coupling to the antenna spatial modes. Further optimization of the device, by reducing the diameter of the hole as well as increasing the depth of the hole by evaporating a thicker Au layer, should further increase the collection efficiency. Overall, the demonstrated approach to back-excitation and the successful direct coupling to a low NA fiber, in particular, point the way to practical platforms for future fiber-coupled on-chip RT SPS systems, which are needed for envisioned compact, networked quantum communication systems,^26,51−56^ with quantum key distribution (QKD) networks employing the wavelength division multiplexing method in particular.

## Supporting Information Available

Fabrication and characterization methods including figures of QD performance, fiber coupling efficiency calculation, and loss estimates (PDF)
