# Supplementary material for: Room-Temperature Fiber-Coupled Single-Photon Sources based on Colloidal Quantum Dots and SiV Centers in Back-Excited Nanoantennas
Source: Nano Lett. 2024 Jan 2;24(2):640–8. doi: 10.1021/acs.nanolett.3c03672 (PMC11139382; doi:10.1021/acs.nanolett.3c03672)
Supplement: Supplementary file 1 — nl3c03672_si_001.pdf [file nl3c03672_si_001.pdf]

Supporting Information for "Room-Temperature Fiber-Coupled Single-Photon Sources based on Colloidal Quantum Dots and SiV Centers in Back-Excited Nanoantennas"

Boaz Lubotzky,[†] Alexander Nazarov,[†] Hamza Abudayyeh,[†] Lukas Antoniuk,[¶] Niklas Lettner ,[¶] Viatcheslav Agafonov ,[‖] Anastasia V. Bennett,[⊥] Somak Majumder,[⊥] Vigneshwaran Chandrasekaran,[⊥] Eric G. Bowes,[⊥] Han Htoon,[⊥] Jennifer A. Hollingsworth,[⊥] Alexander Kubanek,[¶] and Ronen Rapaport[*,†]

†*Racah Institute of Physics, The Hebrew University of Jerusalem, Jerusalem 9190401, Israel*

‡*The Center for Nanoscience and Nanotechnology, The Hebrew University of Jerusalem, Jerusalem 9190401, Israel*

¶*Institute for Quantum Optics, University of Ulm, Albert-Einstein-Allee 11,89081 Ulm, Germany*

§*Center for Integrated Quantum Science and Technology (IQst), Ulm University, Albert-Einstein-Allee 11, D-89081 Ulm, Germany*

‖*GREMAN, UMR 7347 CNRS, INSA-CVL, Tours University, 37200 Tours,France*

⊥*Materials Physics & Applications Division: Center for Integrated Nanotechnologies, Los Alamos National Laboratory, Los Alamos, New Mexico 87545, USA*

E-mail: ronenr@phys.huji.ac.il

Appendix A - Design and fabrication of the nano-antenna devices

We used the template stripping method in order to fabricate and achieve high quality bullseye antennas, as reported previously.[1,2] With this method, after the stripping we will be left with a device that consists of a sapphire slide on top of which are two transparent layers - SU8 3010 photo-resist with a thickness of $\sim$ 10 microns, and on top a layer of gold with a thickness of 250 nm. We used a Focused Ion Beam (FIB) machine to drill a hole in the center of the antenna. The FIB allowed us to drill a hole in a controlled manner so that it was possible to detect when the gold layer was removed and immediately stop. In this way we could remove the gold layer and be left with only the two bottom layers which are transparent. For a hole with a diameter of 400 nm we used a 30 kV ion beam with 7.7 pA current.

In order to determine the desired size of the hole, we used a Lumerical simulation. As mentioned in the main text, the hole size must be large enough to allow transmission of the laser light through the hole and excite the dipole, and small enough to maintain the CE by reducing the transmission of the light emitted from the dipole source. For this purpose, we performed a simulation of the laser transition and the CE as a function of the hole diameter, and thus we could choose a size that would match both requirements, as shown in the in Fig. 1d in the main text. The simulation was based on the model described in[3] with the addition of the hole with different diameter values. Before placing the emitter on the antenna the sample was covered with a dielectric layer of aluminum oxide using ALD deposition which allows us to determine with nanometric precision the thickness of the dielectric layer. We measured the thickness of the aluminum oxide layer in the region of the hole and the hole size (d) using AFM as illustrated in the inset of Fig. 1a. The optimized values of our samples, which are shown in Fig. 1a are d =400 (400)nm, D=1260 (1430)nm, Λ=560 (635)nm, h= 215 (243)nm for the CQD (SiV) respectively.

Appendix B - Characterization of gQDs and Positioning by Dip-pen Nanolithography

Prior to integration into bullseye antennas, gQDs were characterized in the solution phase for absorption and photoluminescence (PL) properties (Figure S1a, b). Steady-state emission spectra, lifetime, and quantum yield (QY) measurements were made using an Edinburgh Instruments FLS1000 fluorescence spectrometer equipped with 450 W xenon lamp (steady-state emission spectra and QY measurements) and AGILE supercontinuum laser (lifetime measurements) excitation sources. Absorption measurements were made with a Cary 5000 UV-Vis-NIR spectrometer. PL QY was determined using a standard Edinburgh Instruments integrating sphere. QY measurements on hexane dispersions of CdSe/CdS gQDs in quartz cuvettes were made in triplicate, and absorbance spectra were acquired on all samples to confirm that the absorbance value at the excitation wavelength was $<$ 0.1 to limit self-absorption effects. The sample was excited at a wavelength of 490 nm with a monochromator grating of 6.00 nm, and data were collected with an emission monochromator grating of 0.1 nm. The resulting QYs obtained over three measurements afforded a value of 77 ± 2%. PL lifetime measurements were made at an excitation wavelength of 400 nm with a pulse repetition rate of 1 MHz, and the detection wavelength was set to 650 nm (center of the gQD emission signal). Excitation and emission monochromator gratings were set to 3.00 nm and 1.00 nm, respectively. The resulting PL decay curve is shown in Fig. S1b.

The technique used to place individual gQDs into the holes located at the center of the bullseye structures is known as dip-pen nanolithography (DPN). It allowed us to transfer gQDs that are suspended in a high-boiling solvent (dichlorobenzene) and wicked onto the tip of an atomic force microscopy (AFM) cantilever from the AFM tip to the target substrate. The general method was described previously in Ref.,[2] and modified slightly here as follows. The AFM tips were placed in the gQD suspension for $\sim$30 s, and excess

ink was removed prior to writing by conducting a series of quick scans on a region of the substrate well away from the bullseye structures until ink could no longer be seen coming off of the tip in the DPN's 10x optical microscope. The employed tip-surface contact times for writing into the hole containing bullseyes was 0.20 - 0.25 s. While it is certain that the writing tip successfully targeted the bullseye center in each placement attempt, it was not possible to determine whether the tip made contact within the hole or at the top surface or hole edge. Nevertheless, in each of four attempts gQDs were successfully delivered into a hole, with 3 receiving single - gQDs and 1 a small cluster of gQDs. By stating this, we do not mean to imply that DPN can deliver small numbers of nanocrystals with 100% certainty, as the attempted depositions are too few from which to draw such a conclusion. In our previous work studying hole-free bullseyes, we demonstrated a 25% success rate for depositing either a single gQD or a small cluster,[2] and it remains to be determined whether the hole itself plays a role in directing fluid movement on the surface of the device, essentially assisting in the placement process.

After placement into the antennas, we immediately characterized the deposited gQDs using a combination of single-emitter PL spectroscopy, PL lifetime evaluation, and $g^{(2)}(0)$ determination (Fig. S1 c-h). This allowed us to confirm the success of our deposition, including whether single or multiple gQDs were deposited. Specifically. a picosecond pulsed laser (Picoquant LDH-D-C-405) with a wavelength of 405 nm and pulse width of 56 ps was used for excitation. The laser was reflected through a dichroic beamsplitter (Semrock Di02-R405) and then focused onto the sample to a diffraction limited spot size using a 50×, 0.7 NA Olympus objective microscope (LCPLFLN50xLCD), which was used to both excite the sample and collect the PL. Collected photons after passing through the same dichroic beamsplitter and a 590 nm long-pass filter either go to a spectrometer + CCD (Acton SP2300i, pylon100) or a Hanbury Brown-Twiss setup consisting of a 50:50 beamsplitter and two single photon avalanche photodiodes (Excelitas SPCM-AQRH-14). TRPL was analyzed using a TCSPC module (Picoquant Hydraharp 400). Excitation power

before the objective is about 500 nW.

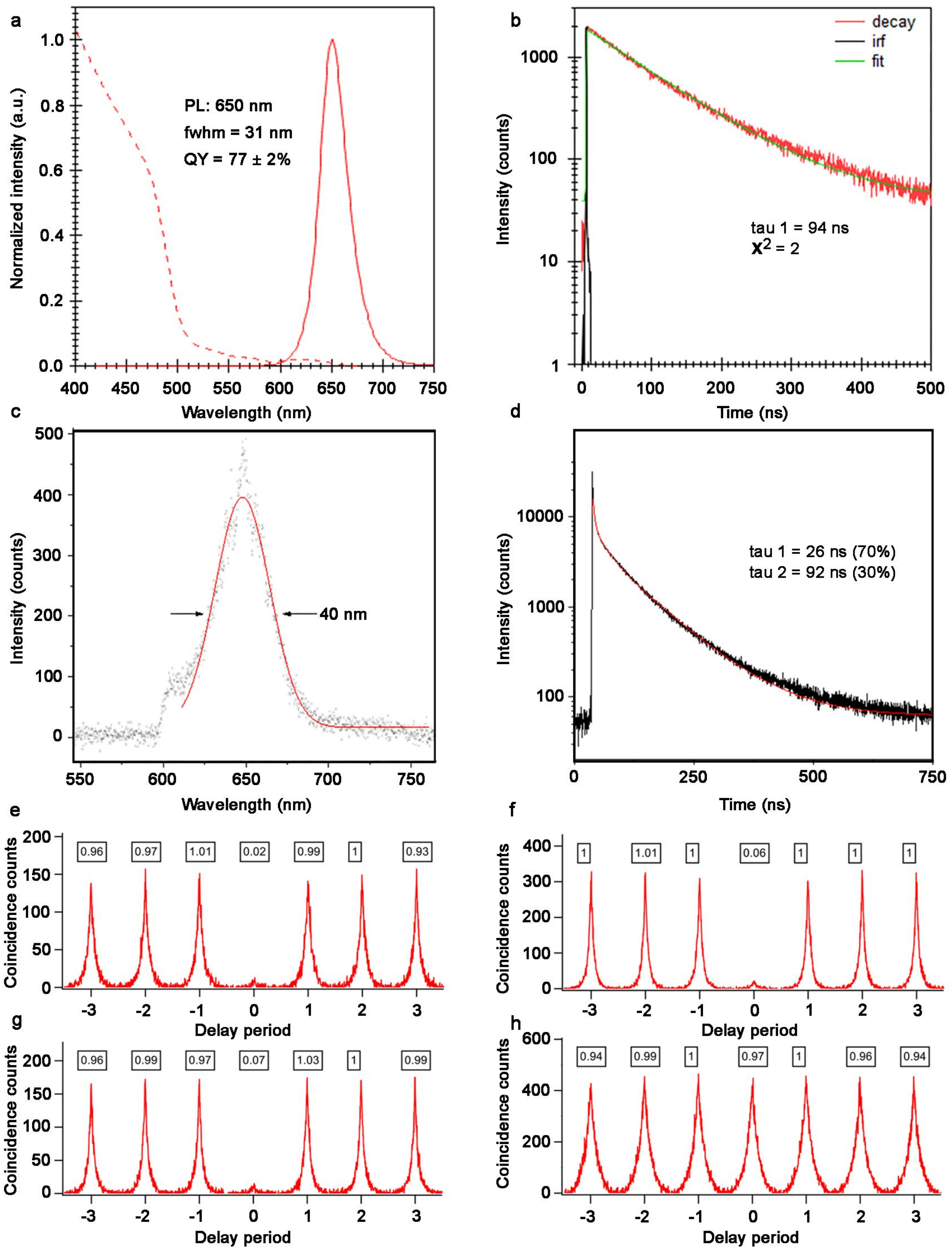

Figure S1: (a) Absorption and emission spectra and (b) PL decay lifetime obtained from gQDs suspended in hexanes. (c) PL spectrum for a single gQD embedded in a bullseye antenna. (d) PL decay lifetime for the single gQD in (c). (e) second-order fluorescence intensity correlation ($g^{(2)}$) for the single gQD in (c). (f) and (g) Correlation data for two other single-gQD/antenna couples. (h) Correlation data for a gQD cluster/antenna couple. In (e)-(h) a time gating of 50 ns was employed to remove contributions from faster biexciton emission (compared to slower exciton emission). Correlation data were used after deposition by dip-pen nanolithography to assess whether single or multiple gQDs were placed. Since $g^{(2)}(0)$ is still large after time-gating for the gQD-antenna device in (h), multiple gQDs are understood to be present.

Appendix C - Preparation and positioning of Nanodiamonds using Pick-and-Place

In preparation of transferring SiV hosting NDs to the bullseye antenna, a dispersion of SiV containing NDs was drop-casted onto a fused silica coverslip. The NDs were fabricated using high-pressure high temperature (HPHT) treatment of the catalyst metals-free hydrocarbon growth system. The NDs were treated with HNO_3 + $HClO_4$ + H_2SO_4 and HF to remove sp2 carbon and were washed and dried afterwards. FIB milled marker structures on the fused silica coverslip serve as a position reference to later locate specific SiV-containing NDs with an AFM. To find SiV-hosting NDs the sample is examined using a homebuilt confocal microscope, orchestrated based on the open-source software qudi.[4] A 2D galvo scanning mirror, a 4f-system and a 1.35 NA oil objective form the basis of this optical setup. Narrowband filtering (740 ± 13 nm) around the ZPL of the SiV increases the signal to noise ratio when exciting offresonantly with 532 nm, as the dominant emission originates from SiV centers within this filter window. After suitable NDs have been located, AFM imaging of the area of interest is carried out. Triangulation of the ND position is done with the help of the FIB markers, as well as positions of other fluorescing NDs in the confocal image. In order to pick and place[5] the ND of interest, a platin-coated AFM cantilever is approached with a constant force until the ND attaches to the tip. The pick-up is indicated by either the disappearing ND in a subsequent non-contact AFM scan or by image artifacts such as 'double-tip' features. Those features also hint towards a successful placement strategy of the ND in the following step. With the ND attached to the cantilever tip, the sample is exchanged for the target sample, here the bullseye antenna. A careful non-contact imaging reveals the target position on the structure. For the antenna structure considered herein the central hole in the structure eases the placement of the ND due to its topology. A contrast in height is useful to detach the ND from the cantilever. After successful ND placement (compare Fig. 3b), the sample is again examined in the

confocal microscope, with the objective being exchanged to 0.55 NA for the sake of a longer working distance and to leverage the collection efficiency.[6] The verification of a successful SiV hosting ND placement is seen in Fig.3c.

Appendix D - Estimation of in-fiber collection efficiency

To estimate the in-fiber collection efficiency, we calculated the fraction of collected photons from all emitted photons using back excitation. First, using the back focal plane image(Fig. 5d), the relative collection efficiency into NA = 0.22 (which is the NA of our MM fiber) was calculated to set the upper limit of the achievable coupling using this device.

$$\frac{\eta_{NA=0.22}}{\eta_{NA=0.9}} = \frac{\int_0^{\theta_{NA=0.22}} d\theta sin(\theta) \int_0^{2\pi} I(\theta,\phi)d\phi}{\int_0^{\theta_{NA=0.9}} d\theta sin(\theta) \int_0^{2\pi} I(\theta,\phi)d\phi} \tag{1}$$

Where η is the collection efficiency and $I(\theta,\phi)$ is the intensity in the back focal plane image. we find

$$\eta_{NA=0.22} = 0.32 \qquad \eta_{NA=0.9} = 0.91 \tag{2}$$

and thus,

$$\frac{\eta_{NA=0.22}}{\eta_{NA=0.9}} = 0.3516 \tag{3}$$

Hence, 35.19% of the light collected by our 0.9 NA objective is emitted at angles smaller than $\theta_{NA=0.22}$. Next, to estimate the experimental coupling efficiency, we compared the counts obtained on a Pixis spectrometer camera for the whole emission spectral band using a collection with a 0.22 NA fiber and a 0.9 NA objective.

$$\frac{I_{Fiber\ Coupled}}{I_{0.9NA\ objective}} = \frac{\int_{\lambda=550nm}^{\lambda=700nm} I_{Fiber\ Coupled}(\lambda)d\lambda \cdot \gamma_{Fiber}^{-1}}{\int_{\lambda=550nm}^{\lambda=700nm} I_{0.9NA\ objective}(\lambda)d\lambda \cdot \gamma_{Objective}^{-1}} \tag{4}$$

where the integral represents the area under the spectrum and γ stands for the total measured loss in each collection setup (free space and fiber collection setups). The

losses in the objective system are divided into two distinct types:

$$\gamma_{objective} = \gamma_{system\,loses} \cdot \gamma_{spectrometer\,slit} \tag{5}$$

where the losses in the system,$\gamma_{system\,loses}$, are losses caused by the optical system itself (partial transmission of mirrors and filters) and losses caused by the spectrometer's entrance slit,$\gamma_{spectrometer\,slit}$. Since the entrance slit is slightly smaller than the entire emission spatial beam, only a portion of the light that reaches the rectangular slit is transmitted. To calculate the fraction of transmitted light, we use the following expression:

$$\gamma_{spectrometer\,slit} = \frac{\Gamma w}{\pi \cdot \Gamma^2} \tag{6}$$

Where Γ is the FWHM of the Gaussian emission profile on the spectrometer camera along the slit axis, and w is the slit width. To calculate the FWHM of the power distribution, we sum the intensities along the camera and not along the grating. As seen in the figure below S2 the FWHM is 44 pixels.

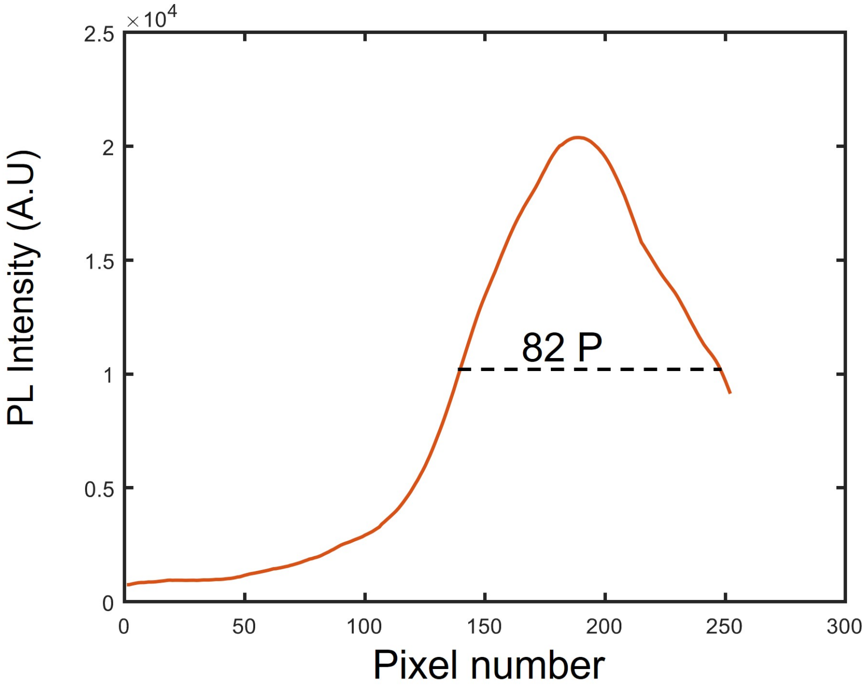

Figure S2: FWHM of the intensity distribution on the spectrometer camera

Since the pixel size is 82 microns, the FWHM is $2132\,\mu\mathrm{m}$. By using the known width of the spectrometer slit which is $500\,\mu\mathrm{m}$, we can substitute it into 6:

$$\gamma_{spectrometer\ slit} = \frac{2132\,\mu\mathrm{m} \cdot 500\,\mu\mathrm{m}}{\pi \cdot (2132\,\mu\mathrm{m})^2} \approx 0.074 \tag{7}$$

To determine the loss coefficient for the objective system, we used a He:Ne laser. By measuring the power before the objective and after all the mirrors and filters, we found that the measured power was $6.65\,\mu\mathrm{W}$ and $17\,\mu\mathrm{W}$, respectively. Therefore the loss coefficient for the objective system is approximately $\gamma_{system:loses} \approx 0.3911$.

The losses in the fiber coupling system were calculated using the same He:Ne laser. The intensity of the laser was measured before the entrance to the fiber. After the filters on the other side of the fiber, the intensity before the entrance was $17\,\mu\mathrm{W}$ and $14.6\,\mu\mathrm{W}$ at the end of the system (after all of the mirrors and filters). Hence, the loss of the fiber system was $\gamma_{fiber\ loss} \approx 0.859$. Here again, we must find the loss due to the spectrometer

slit.

$$\gamma_{Fiber} = \gamma_{fiber\ system} \cdot \gamma_{fiber\ spectrometer\ slit} \tag{8}$$

Again, we will find the FWHM of the intensity distribution on the camera. As it can be seen, the FWHM is 43 pixelsS3; multiplying that by the size of the pixels, we get 1118 microns.

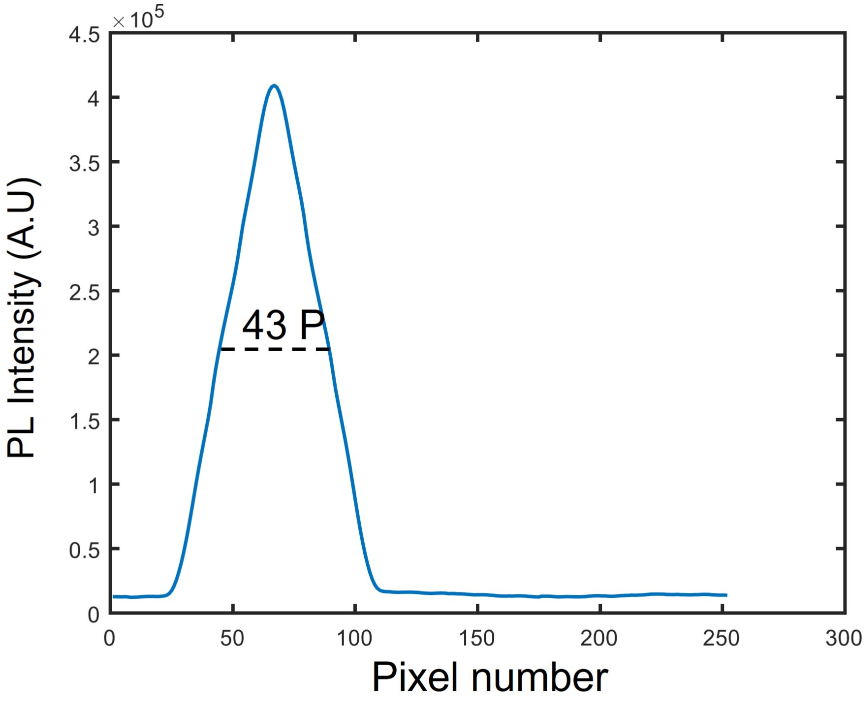

Figure S3: fiber measurement: FWHM of the intensity distribution on the spectrometer camera

$$\gamma_{spectrometer\ slit} = \frac{\Gamma w}{\pi \cdot \Gamma^2} = \frac{500 \cdot 1118}{\pi \cdot (1118)^2} = 0.1423 \tag{9}$$

and hence,

$$\gamma_{Fiber} = 0.0854 \cdot 0.859 \approx 0.1222 \tag{10}$$

Now substituting all the values into the previous equation 4.

$$\frac{I_{Fiber\ Coupled}}{I_{0.9NA\ objective}} \approx \frac{\int_{\lambda=550nm}^{\lambda=700nm} I_{Fiber\ Coupled}(\lambda) d\lambda \cdot 8.177}{\int_{\lambda=550nm}^{\lambda=700nm} I_{0.9NA\ objective}(\lambda) d\lambda \cdot 34.25} = \frac{98300 \cdot 8.177}{237500 \cdot 34.25} \approx 0.0988 \tag{11}$$

And hence the effective coupling efficiency is $9.88 \pm 0.48\%$.

Appendix E - Spectrum broadening in-fiber measurmets

Spectral broadening is evident upon comparison of the front excitation, back excitation, and fiber collection spectra in Fig. 5c. While the QD emission remained unchanged, the primary contribution to the observed broadening of the back excited QDs was broadband plasmon emission from the metal resulting from the varying excitation laser powers utilized for each configuration. To compensate for optical laser back coupling losses, the excitation power for free space back excitation experiment was 10 times higher compared to front excitation. Further, excitation power for the back excited fiber coupling was 75 times higher than for front excitation, required to overcome fiber insertion losses. The increased powers enhance plasmon emission, decreasing the quantum yield. This interpretation is supported by broadband visible to near-IR fluorescence from the unoccupied antenna under high power back excitation (Fig. S4).

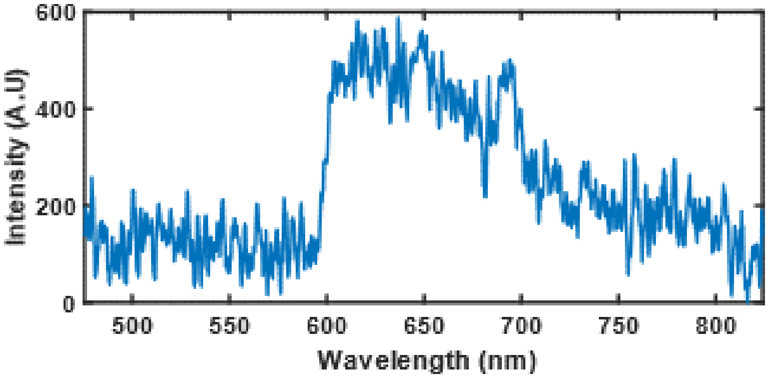

Figure S4: Spectrum measurement of an empty antenna with a long pass filter at 600nm

References

(1) Abudayyeh, H.; Mildner, A.; Liran, D.; Lubotzky, B.; Lüder, L.; Fleischer, M.; Rapaport, R. Overcoming the Rate-Directionality Trade-off: A Room-Temperature Ultrabright Quantum Light Source. *ACS Nano* **2021**,

(2) Abudayyeh, H.; Lubotzky, B.; Blake, A.; Wang, J.; Majumder, S.; Hu, Z.; Kim, Y.; Htoon, H.; Bose, R.; Malko, A. V.; Hollingsworth, J. A.; Rapaport, R. Single photon sources with near unity collection efficiencies by deterministic placement of quantum dots in nanoantennas. *APL Photonics* **2021**, *6*.

(3) Abudayyeh, H.; Rapaport, R. Quantum emitters coupled to circular nanoantennas for high brightness quantum light sources. *Quantum Sci. Technol.* **2017**, 1–25.

(4) Binder, J. M.; Stark, A.; Tomek, N.; Scheuer, J.; Frank, F.; Jahnke, K. D.; Müller, C.; Schmitt, S.; Metsch, M. H.; Unden, T.; Gehring, T.; Huck, A.; Andersen, U. L.; Rogers, L. J.; Jelezko, F. Qudi: A modular python suite for experiment control and data processing. *SoftwareX* **2017**, *6*, 85–90.

(5) Schell, A. W.; Kewes, G.; Schröder, T.; Wolters, J.; Aichele, T.; Benson, O. A scanning probe-based pick-and-place procedure for assembly of integrated quantum optical hybrid devices. *Review of Scientific Instruments* **2011**, *82*, 073709.

(6) Waltrich, R.; Lubotzky, B.; Abudayyeh, H.; Steiger, E. S.; Fehler, K. G.; Lettner, N.; Davydov, V. A.; Agafonov, V. N.; Rapaport, R.; Kubanek, A. High-purity single photons obtained with moderate-NA optics from SiV center in nanodiamonds on a bullseye antenna. *New Journal of Physics* **2021**, *23*.
